# Short-chain fatty acids are a key mediator of gut microbial regulation of T cell trafficking and differentiation after traumatic brain injury

**DOI:** 10.21203/rs.3.rs-5397327/v1

**Published:** 2024-11-21

**Authors:** Marta Celorrio, Kirill Shumilov, Allen Ni, Wade K. Self, Francisca N. L. Vitorino, Rachel Rodgers, Lawrence A. Schriefer, Ben Garcia, Brian T. Layden, Gabor Egervari, Megan T. Baldridge, Stuart H. Friess

**Affiliations:** Washington University in St. Louis School of Medicine; Virginia Commonwealth University; Washington University in St. Louis School of Medicine; Washington University in St. Louis; Washington University in St. Louis School of Medicine; Washington University in St. Louis School of Medicine; Washington University in St. Louis School of Medicine; Washington University in St. Louis School of Medicine; University of Illinois at Chicago; Washington University in St. Louis School of Medicine; Washington University in St. Louis School of Medicine; Washington University in St. Louis School of Medicine

**Keywords:** Traumatic brain injury, gut microbiota depletion, gut microbiome, short-chain fatty acids, T cells, microglia, neurogenesis, T cell-trafficking, neuroinflammation, gut-brain axis

## Abstract

The gut microbiota has emerged as a pivotal regulator of host inflammatory processes after traumatic brain injury (TBI). However, the mechanisms by which the gut microbiota communicates to the brain in TBI are still under investigation. We previously reported that gut microbiota depletion (GMD) using antibiotics after TBI resulted in increased microglial activation, reduced neurogenesis, and reduced T cell infiltration. In the present study, we have demonstrated that intestinal T cells contribute to the pool of cells infiltrating the brain after TBI. Depletion or genetic deletion of T cells before injury reversed GMD induced reductions in post-TBI neurogenesis. Short-chain fatty acid supplementation increased T regulatory and T helper1 cell infiltration to the brain along with restoring neurogenesis and microglia activation after TBI with GMD. These data suggest that T cell subsets are essential cellular mediators by which the gut microbiota modulates TBI pathogenesis, a finding with important therapeutic implications.

## Introduction

Traumatic brain injury (TBI) remains a global public health concern with survivors facing short and long-term disabilities(1). Approximately 1.7 million Americans experience TBI each year, with more than 5 million people impacted by TBI-related disabilities(2). Despite advances in care and research, gaps persist in understanding the full scope of the disease burden, and specifically in enhancing treatment to achieve better outcomes. Neuroinflammation after TBI impacts injury severity and recovery, and its dysregulation may contribute to neurodegeneration and cognitive impairments observed in some TBI survivors(3, 4). Furthermore, neuroinflammation also plays an important role in tissue repair and remodeling post-injury(5). TBI also impacts the gastrointestinal tract, inducing gut microbial depletion (GMD) in humans that worsens long-term morbidity and mortality(6). Recent investigations into the gut-brain axis in the setting of TBI have focused on the impact of TBI on gut function (dysmotility and increased mucosal permeability), the gut microbiota (changes in richness and diversity)(7), and behavior(8). Additionally, TBI not only influences local neuroinflammation in the brain but also has a profound impact upon the peripheral immune response. We previously found that GMD induces alterations in the central and peripheral immune responses, reduces post-TBI neurogenesis and worsens outcomes after TBI. However, our understanding of the mechanisms by which the gut communicates to the brain and how it modulates injury severity and recovery after TBI is still limited.

Recent evidence suggests that T cells play an important role in the regulation of immune responses during chronic inflammation after TBI, impacting recovery(9, 10). Upon activation, T cells release proinflammatory cytokines such as interleukins (IL17) or interferon gamma (IFNγ), interacting with resident immune cells (microglia) and amplifying the inflammatory response(3). However, T regulatory cells (T reg) play a role in regulating these immune responses and suppressing excessive inflammation after TBI(11). Therefore, shifting from pro-inflammatory to anti-inflammatory T cell responses may improve recovery and neurological outcomes following TBI. We and others have found that T cell responses after brain injury such as TBI and stroke are influenced by GMD(12–14). This suggests that gut bacteria can modulate not only gut-resident T cells but also their access and response in the brain and their interactions with brain-resident immune cells(15).

A growing body of literature supports that TBI induces alterations of both specific bacterial groups (16) and their metabolites, including short-chain fatty acids (SCFAs)(17). SCFAs, through both activation of G-protein-coupled receptors (GPCRs) and inhibition of histone deacetylases (HDAC), are essential for regulation of neuroinflammation(18), blood brain barrier (BBB) permeability(19), and stress behavior(20). SCFAs can cross the BBB and affect brain function, influencing neuronal activity and neuroinflammatory responses(21). In addition, SCFAs can also enhance T cell responses from the gut, which can indirectly affect CNS inflammation after brain injury(22, 23). In light of these findings, we hypothesize that SCFAs influence both brain-resident and peripheral immune responses towards more anti-inflammatory phenotypes to reduce the severity of outcomes after TBI with GMD.

To date, the possible mechanism and impact of gut communication to the brain in the setting of TBI is still under investigation. We hypothesize that T cell differentiation and infiltration and into the brain after TBI can be regulated by the gut microbiota and modulate the neuroinflammatory responses impacting neurogenesis and microglia function. In this paper, we aim to address a critical gap in knowledge by utilizing a mouse model of TBI with manipulation of the gut microbiota to dissect out the immunological mechanisms by which the gut communicates with the brain after TBI. Our study provides important insights into further development of therapies targeting T cells in conjunction with SCFAs to modulate the onset and progression of secondary injuries after TBI.

## Materials and methods

### Animals

All procedures were approved by the Washington University Animal Studies Committee and are consistent with the National Institutes of Health guidelines for the care and use of mice. Mice were housed 5/cage and had free access to water and food with a 12-hour light/dark cycle. Wild-type (WT) C57BL/6J (RRID: IMSR_ORNL:C57BL/6J-A/A) were purchased from Jackson Laboratory (Bar Harbor, ME). B6.129P2-Tcrb^tm1Mom^Tcrd^tm1Mom/J^J (TCRb^−/−^TCRd^−/−^) (RRID:IMSR_JAX:002122) were purchased from Jackson Laboratory (Bar Harbor). *Gpr43*^−/−^ mice (owned by Northwestern University) and *Gpr41*^−/−^ mice were donated from the laboratory of Megan Baldrigde. Tg(CAG-KikGR)33Hadj/J (KikGR, JAX 013753) KikGR33 mice were donated from the laboratory of Jonathan Kipnis. All the mice were 8-week-old male and female mice weighing 20–25 grams (g) were used for *in vivo* studies.

### Controlled cortical impact with gut microbial depletion.

Controlled cortical impact (CCI) was performed using a previously described protocol(13). Mice were anesthetized with 5% isoflurane and maintained at 2% isoflurane throughout the procedure. Buprenorphine sustained-release (0.5 mg/kg) was administered subcutaneously, prior to scalp incision. Ear bars were positioned to secure the head within the stereotaxic frame (MyNeurolab, St. Louis, MO). A 5 mm craniectomy was performed using an electric drill, centered 2.7 mm lateral to the midline and 3 mm anterior to lambda. The electronic impactor (Leica Biosystems, Richmond, VA) equipped with a 3 mm tip was aligned with the craniectomy site using the following coordinates: 1.2 mm lateral to the midline and 1.5 mm anterior to lambda(13). The impact was delivered at a depth of 2 mm with a velocity of 5 m/s and a dwell time of 100 ms.

A loose-fitting 7 mm plastic cap was secured over the craniectomy site using Vetbond (3M, St. Paul, MN). The skin incision was closed with interrupted sutures and treated with antibiotic ointment. Mice were then placed on a warming pad for recovery. For the sham conditions, a skin incision procedure was performed to serve as a control. This involved making an incision without any further surgical intervention, allowing us to isolate any possible effects caused by the surgical process itself.

For gut microbiota depletion, broad-spectrum antibiotics were administered for 7 consecutive days in the drinking water consisting of 250 mg vancomycin, 500 mg neomycin sulfate, 500 mg ampicillin, 500 mg metronidazole (VNAM), and 20 g grape-flavored Kool-Aid (Kraft Heinz, Chicago, IL) in 500 mL of sterile-filtered water(24). Control mice received only Kool-Aid in drinking water. Cages and bottles were autoclaved prior to use.

### SCFA supplementation

SCFA supplementation was a mix of 25 mM sodium propionate, 40 mM sodium butyrate and 67.5 mM sodium acetate (Sigma-Aldrich) added to VNAM or Kool-Aid drinking water solution for 1 week.

### SCFA quantification by mass spectrometry

SCFAs were measured in the stool and plasma. Briefly, stool samples were homogenized in phosphate-buffered saline (PBS) (15 mL PBS/g stool). The 5-point standard curves for acetic acid, propionic acid, and butyric acid were prepared in phosphate-buffered saline (PBS). To the 20 μL of stool homogenate or plasma was added internal standards (d4-AA, d2-PA, d3-BA), followed by acidification, neutralization, and derivatization with aminomethyl phenyl pyridinium (AMPP) and 1-ethyl-3-(3-dimethylamino propyl)carbodiimide hydrochloride (EDC). The volatile short-fatty acids and their internal standards were converted into non-volatile AMPP amides. A protein precipitation procedure was used to extract AMPP derivatives of SCFAs and internal standards from reaction mixture. The extracts were separated by column-switching high-performance liquid chromatography (HPLC) on a SecurityGuard C18 (4 × 3 mm) and ACE 3 C18 column (3μm, 150 × 4.6 mm). Fatty acid derivatives and their internal standards were monitored by an Applied Biosystems Sciex 4000QTRAP tandem mass spectrometer (MS/MS) equipped with an electrospray ion source in the positive ion mode and multiple-reaction monitoring (MRM) detection.

### Fecal bacterial analysis

Fecal pellets were collected into sterile 1.7 ml tubes. Phenol:chloroform-extracted DNA from fecal pellets was used for 16S rRNA gene sequencing. For 16S sequencing, primer selection and PCRs were performed as described previously(25). Briefly, each sample was amplified in triplicate with Golay-barcoded primers specific for the V4 region (F515/R806), combined, and confirmed by gel electrophoresis. PCR reactions contained 18.8 μL RNase/DNase-free water, 2.5 μL 10X High Fidelity PCR Buffer (Invitrogen, 11304–102), 0.5 μL 10 mM dNTPs, 1 μL 50 mM MgSO4, 0.5 μL each of the forward and reverse primers (10 μM final concentration), 0.1 μL Platinum High Fidelity Taq (Invitrogen, 11304–102) and 1.0 μL genomic DNA. Reactions were held at 94°C for 2 min to denature the DNA, with amplification proceeding for 26 cycles at 94°C for 15 s, 50°C for 30 s, and 68°C for 30 s; a final extension of 2 min at 68°C was added to ensure complete amplification. Amplicons were pooled and purified with 0.6x Agencourt AMPure XP beads (Beckman-Coulter, A63882) according to the manufacturer’s instructions. The final pooled samples, along with aliquots of the three sequencing primers, were sent to the DNA Sequencing Innovation Lab (Washington University School of Medicine) for sequencing by the 2X 250 bp protocol with the Illumina MiSeq platform. Read quality control and the resolution of amplicon sequence variants were performed with the dada2 R package(26). Amplicon sequence variants that were not assigned to the kingdom Bacteria were filtered out. The remaining reads were assigned taxonomy using the Ribosomal Database Project (RDP trainset 16/release 11.5) 16S rRNA gene sequence database(27). Ecological analyses, were performed using PhyloSeq and additional R packages(28).

### Histone extraction

Histones were extracted as previously described(29). Injured brain tissue was homogenized in nuclear isolation buffer (15 mM Tris-HCl, 15 mM NaCl, 60 mM KCl, 5 mM MgCl_2_, 1 mM CaCl_2_, and 250 mM sucrose at pH 7.5; 0.5 mM AEBSF, 10 mM sodium butyrate, 5 nM microcystein, and 1 mM DTT added fresh) with 0.3% NP-40 and incubated on ice for 5 min. The nuclei were collected by centrifuging at 600 g at 4°C for 5 min. The resulting nuclear pellet was washed twice with the same volume of nuclear isolation buffer without NP-40. Histones were then acid-extracted with 0.2 N H_2_SO_4_ for 2 h at 4°C with rotation. The insoluble nuclear debris was pelleted at 3,400 g at 4°C for 5 min, and the supernatant was retained. Next, histone proteins were precipitated by adding 100% trichloroacetic acid in a 1:4 ratio (v/v) O/N at 4°C. The pellet was washed with acetone to remove residual acid. Histones were resuspended in 30 μl of 50 mM NH_4_HCO_3_ (pH 8.0).

### Histone propionylation and digestion

Histones were derivatized and digested as previously described(29). Injured brain tissue was mixed with 15 μl derivatization mix, consisting of propionic anhydride and acetonitrile in a 1:3 ratio (v/v), and this was immediately followed by the addition of 7.5 μl ammonium hydroxide to maintain pH 8.0. The sample was incubated for 15 min at room temperature (RT), and the derivatization procedure was repeated one more time. Samples were then dried and resuspended in 50 mM NH_4_HCO_3_ and incubated with trypsin (enzyme:sample ratio of 1:20) overnight at RT. After digestion, the derivatization reaction was performed again twice to derivatize the N termini of the peptides. Samples were desalted using C18 stage tips before liquid chromatography with tandem mass spectrometry (LC-MS/MS) analysis.

### LC-MS/MS analysis

An LC-MS/MS system consisting of a Vanquish Neo UHPLC coupled to an Orbitrap Q Exactive or Ascend (Thermo Scientific, Waltham, MA) was used for peptide analysis. Histone peptide samples were maintained at 7°C on a sample tray in LC. Separation of peptides was carried out on an Easy-Spray^™^ PepMap^™^ Neo nano-column (2 μm, C18, 75 μm X 150 mm) at RT with a mobile phase. The chromatography conditions consisted of a linear gradient from 2 to 32% solvent B (0.1% formic acid in 100% acetonitrile) in solvent A (0.1% formic acid in water) over 48 min and then 42 to 98% solvent B over 12 min at a flow rate of 300 nL/min. The mass spectrometer was programmed for data-independent acquisition (DIA). One acquisition cycle consisted of a full MS scan and 35 DIA MS/MS scans of 24 m/z isolation width starting from 295 m/z to 1100 m/z. Typically, full MS scans were acquired in the Orbitrap mass analyzer across 290–1200 m/z at a resolution of 70,000 or 120,000 in positive profile mode with an injection time of 50 ms and an AGC target of 1e6 or 200%. MS/MS data from HCD fragmentation was collected in the Orbitrap. These scans typically used an NCE of 30 or 25, an AGC target of 1000%, and a maximum injection time of 60 ms. Histone MS data were analyzed with EpiProfile(30).

### CD3-especific monoclonal antibody injection

For *in vivo* depletion of CD3 expressing cells, mice were injected intraperitoneally (i.p.) with 200 μg of InVivoPlus anti-mouse CD3ε, clone 145–2C11 (1 μg/μl, Cat# BP0001-1, Bio X Cell, Lebanon, New Hampshire), starting 6 days before injury, followed by 100 μg injections every four days for 1 month. The control group received the same injection regime with InVivoPlus polyclonal Armenian hamster IgG (Cat# BP0091 Bio X Cell, Lebanon, New Hampshire).

### 5-bromo-2’-deoxyuridine (BrdU) treatment

To detect cell proliferation in the injured hippocampus, mice received injections of 5-bromo-2’-deoxyuridine (BrdU, Sigma-Aldrich, St. Louis, MO) 50 mg/kg i.p. daily for 4 consecutive days starting 3 days post-injury.

### Cell isolation from brain and blood samples

The blood and the regions of interest in the ipsilateral brain (hippocampus and cortex) were harvested 7 days after injury. Five mice (from all conditions) were processed per day with a total of 20 samples at the time. Mice were anesthetized with isoflurane, and blood samples were taken in EDTA tubes immediately before transcardial perfusion with ice-cold 0.1 M heparinized-PBS. The brain regions of interest were dissected out on ice and digested at 37°C for 15 min with collagenase D (400 units/mL, Roche, Diagnostics Gmbh, Mannheim, Germany) in Dulbecco’s PBS (Lonza, Basel, Switzerland), each containing 50 μg/mL of DNase I (Sigma-Aldrich). The tissue was then mechanically dissociated with a glass Pasteur pipette, filtered through a 70-μm nylon cell strainer, and centrifuged at 950 rpm for 15 min. A 25% Percoll (Sigma-Aldrich) column was used to remove cell debris and myelin, followed by centrifugation at 1700 rpm for 10 min. 25 μl-blood sample was mixed with 1x Red Blood Lysis Buffer (Roche) and incubated in rotation for 15 min at RT. Samples were then centrifuged at 3500 rpm for 5 min at RT. The supernatant was discarded, and cells were washed and resuspended in 1 mL of cytometer buffer [0.5% bovine serum albumin (Sigma-Aldrich), 5 mM EDTA (Millipore, Burlington, MA) in PBS]. Then, the cells were resuspended in 100 μl of cytometer buffer and stained.

### Cell isolation from meninges

Dura mater from the skull was carefully harvested in a RPMI solution under a stereomicroscope as previously described(31). Briefly, dura mater was digested for 30 min at 37°C in prewarmed digestion buffer [RPMI with 1 mg/ml Collagenase VIII (Sigma-Aldrich) supplemented with 0.5 mg/ml of deoxyribonuclease I (DNase I) (Sigma-Aldrich), 2% fetal bovine serum (FBS)]. After digestion, dura mater underwent mechanical homogenization followed by filtration through a 70-μm cell strainer. Enzymes were inactivated with RPMI with 10% fetal bovine serum (FBS), and cells were then centrifuged at 2000 rpm for 10 min and washed in cytometer buffer. Single-cell suspensions were kept in cytometer buffer on ice until use.

### Cell isolation of lamina propia (LP) immune cells

The small intestines were removed and separated. Peyer’s patches were excised, and intestines were cleaned of mesenteric fat and intestinal contents with PBS. Then intestines were opened longitudinally, washed with HBSS/HEPES, cut into 1-cm pieces and placed into 20 ml of HBSS/HEPES 10% bovine calf serum and 5 mM EDTA for 20 min at 37°C. During this period, tissue suspensions were vortexed for 15 s each 5 min. Tissue pieces were washed with HBSS/HEPES to remove EDTA, minced thoroughly, and placed into 10 ml RMPI with 10% FBS, 0.01M HEPES, 1x Kanamycin, 1x Glutamax, 1x Sodium pyruvate, 1x non-essential amino acids, 10 mg/mL DNAase I (Roche), and 100 U/ml collagenase IV (Sigma-Aldrich). Tissues were digested at 37°C for 50 min with constant agitation (250 rpm). The resulting LP cell suspensions were filtered through a 70-μm nylon cell strainer and washed with 10 ml HBSS/HEPES. LP cell suspensions were collected at 2000 rpm for 10 min at 4°C. Cell pellets were resuspended in 4 ml 40% Percoll (GE healthcare, Chicago, IL) and overlaid over 3 ml of 80% Percoll. Gradients were centrifuged at 2000 rpm for 20 min at 4°C and cells at the interface were collected and washed with 50 ml PBS. Cells were stained for flow cytometric analysis.

### Flow cytometry staining and analysis

Cells were incubated for 5 min at RT with Zombie NIR Dye (BioLegend, San Diego, CA) to assess their viability. The Zombie NIR Dye was quenched, and cells were washed with cytometry buffer and blocked with FcR blocking reagent (1:50, Miltenyi Biotec, Bergisch Gladbach, Germany). Then, the samples were washed with cytometry buffer, stained with antibodies (Table 1) for 15 min at RT. Microglial cells were defined as CD45^low^CD11b^+^ and T cells as CD45^hi^CD11b^−^CD3^+^. For the intracellular staining, cells were first stained with surface markers (Table 1), stimulated, fixed and permeabilized by using FoxP3/transcription factor staining buffer set (eBiosciences, Waltham, MA) following the manufacturer’s instructions. Briefly, cells were stimulated for 4 h with 0.2 μg/mL phorbol 12-myristate 13-acetate (PMA), 2 μg/mL ionomycin, and 1x brefeldin A to characterize T cell subset changes associated with GMD after TBI. Next cells were fixed for 7 mins at 4°C, washed, permeabilized and stained with intracellular markers (Table 1) for 30 mins at 4°C. We measured the percentages of TNFα and IL-17 + T cells to quantify the populations of T helper (Th)1 and Th17 cells, respectively. Then, cells were washed and analyzed on a BD LSRFortessa flow cytometer (BD Biosciences, Franklin Lakes, NJ) using the Software v10.6.1 (BD Biosciences, Franklin Lakes, NJ). Fluorescence minus one (FMO) was used as negative controls for each marker.

### T cells isolation from spleen

Splenocytes were obtained by mechanical shredding and filtered through a 70-μm cell strainer, and centrifuged at 500g for 10 min. The resulting cell suspensions were incubated with red blood lysis buffer (Roche) for 5 min at 4°C, centrifuged and filtered through 40-μm cell strainer. T cells were then isolated following manufacturers protocol by negative selection using pan T cell isolation kit II (Miltenyi biotec, Bergisch Gladbach, Germany). This isolation kit is based on a cocktail of biotin-conjugated antibodies against CD11b, CD11c, CD19, CD49b, CD105, Anti-MHC-class II, and Ter-119.

### Photoconversion of KikG33 mice cells

We used KikGR33 transgenic mice express a Kikume Green-Red (KikGR) photoconvertible fluorescent protein that undergoes stable photoconversion from a green to a red form after exposure to violet light(32). Mice were anesthetized with 5% isoflurane and maintained at 2% isoflurane throughout the procedure. Buprenorphine sustained release (0.5 mg/kg) was administered subcutaneously, prior to abdominal incision. Small intestine (SI) photoconversion was performed as previously(12). Immediately prior to CCI, 8-week-old male and female mice underwent laparotomy to expose 6 cm of SI (ileum) under anesthesia. The rest of the mouse was shielded with aluminum foil to avoid off-target photoconversion. The exposed intestines were illuminated with a violet laser source (405 nm, Prizmatix, Washington DC) for 20 mins followed by careful repositioning of the intestines in the abdominal cavity and the peritoneum and skin sutured. The exposed intestines were kept hydrated by applying saline throughout the 20 mins. Seven days after injury, mice were euthanized for brain, blood, meninges, and SI cell isolation and preparation as previously indicated. Flow cytometric analysis of the percentage of photoconverted red cells (KikR^+^ cells) in lymphocytes (CD45^hi^CD11b^−^ cells) and monocytes/macrophages (CD45^hi^CD11b^+^ cells) was done by spectral flow cytometry (Cytek Biosciences, Fremont, CA).

### Tissue Processing

Mice were euthanized under isoflurane anesthesia, by transcardial perfusion with cold 0.3% heparin in PBS followed by 4% paraformaldehyde solution in PBS (PFA, Sigma-Aldrich). Brains were post-fixed in 4% PFA for 24 h at 4°C followed by equilibration in 30% sucrose for 48 h before sectioning. 50-μm thick cryosections were cut using a freezing microtome.

### Fluorescence Immunohistochemistry

Fluorescence immunohistochemical staining was performed on free-floating sections. Tissue was incubated with pre-heated HCl 1N (Sigma-Aldrich) for 30 min at 45°C to increase the antigen exposure for BrdU detection. After the three washes with PBS, 20% normal donkey serum, 3% bovine serum albumin, and 0.3% triton X-100 in PBS were used to block nonspecific staining for all antibodies. Sections with a thickness of 50 μm were stained with the primary antibodies (Table 1) at 4°C overnight. The next day, antibody binding was detected by incubating sections with Alexa Fluor secondary antibody (Table 1) for 2 hours in PBS with 0.3% triton X-100. Sections were mounted on glass slides in PBS, dried, and coverslipped with mounting medium for fluorescence with 4’,6-Diamidino-2-Phenylindole, (DAPI, Thermo Fisher Scientific).

### Quantitative fluorescent immunohistochemistry

Fluorescent images were obtained with a Zeiss Axio Imager Z2 with ApoTome 2 fluorescence microscope with a 20X objective. 20-μm z stacks with an interval of 1 μm were obtained of the ipsilateral hippocampus. Quantification of neuronal proliferation was performed by counting the number of cells that co-localized with BrdU, doublecortin (DCX), and NeuN (neuronal nuclei) staining in 4 slices spaced 300 μm apart by a blinded observer.

### Three-dimensional reconstruction of microglia

We followed a published protocol for microglia morphology analysis(13). 50-μm sections were stained with Iba1 (Table 1) 4°C overnight, followed by Alexa Fluor 488–conjugated secondary antibody (Table 1) staining for 2h. Sections were mounted on glass slides in TBS-X, dried, and coverslipped with mounting medium for fluorescence with DAPI. Imaging was performed on a Zeiss LSM 880 confocal laser scanning microscope (Zeiss, White Plains, NY) using a 20X 0.8 NA objective. Z-stacks were done with 1.00-μm steps in z direction; 1,024 × 1,024-pixel resolution were recorded and analyzed using Imaris software (Bitplane, Concord, MA). Three hippocampal cells from the ipsilateral dentate gyrus were reconstructed per analyzed mouse.

### Statistical analysis

Blinding of investigators to experimental groups was maintained until data were fully analyzed. Data were assessed for normal distribution with the Shapiro-Wilk test and expressed as mean ± SEM. Two-tailed Student’s t-test was used when comparing two conditions or Mann-Whitney U test when data was not normally distributed. For more than two conditions, two-way ANOVA followed by post-hoc Tukey and Newman-Keuls tests were employed. All analysis was performed with GraphPad Prism v9.1.0 (GraphPad software. Boston, MA).

## Results

### Intestinal T-cell reservoirs contribute to the pool of cells that infiltrate the brain after TBI.

**Figure 1:**
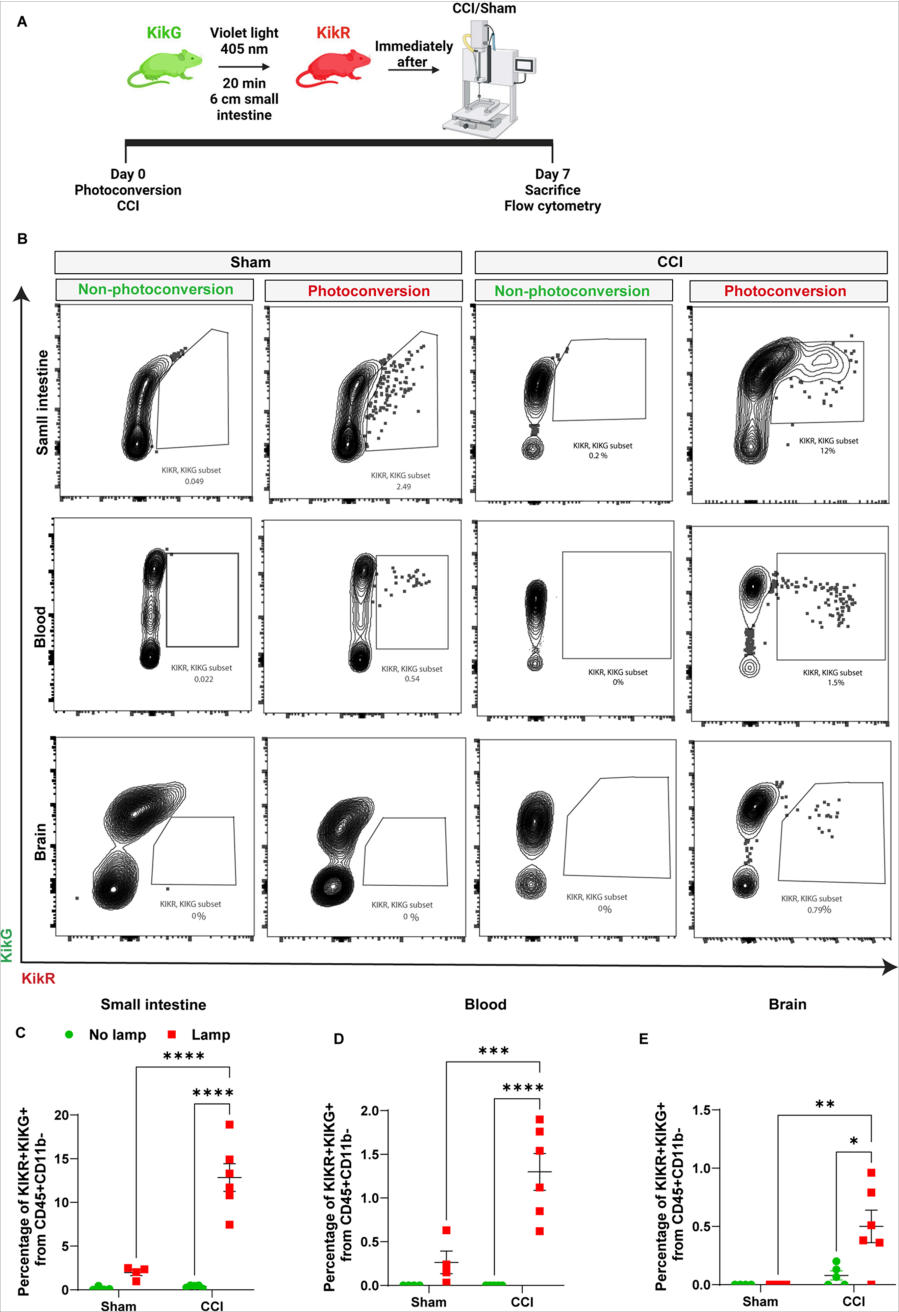
Intestinal T-cell reservoirs contribute to the pool of cells that infiltrate the brain after TBI. A) Experimental design for analysis of KikGR mice. Photoconversion of the distal part of the small intestine is induced using violet light right before CCI or sham, and the photoconverted lymphocytes (CD45^+^CD11b^−^KikR^+^) cells are analyzed in SI, blood, and brain by spectral flow cytometry 7 days after CCI. B) Spectral flow cytometric gating strategy in photoconverted (KikR^+^) mice. Lymphocytes (CD45^+^CD11b^−^) that expresses the red form (KikR^+^) cells 7 days after sham and CCI surgery and with and without photoconversion. Mean values are plotted ± SEM, two-way ANOVA followed by Tukey’s multiple comparisons. F statistic for photoconversion*injury presented unless otherwise noted (*p < 0.05, **p < 0.01 ***p < 0.001, ****p < 0.0001), n = 4–6 per group. C) Frequency of photoconverted lymphocytes (CD45^+^CD11b^−^KikR^+^) in the small intestine (lamina propia) F_(1, 15)_ = 25.43, p=0.0001, D) blood F_(1, 15)_= 12.14, p=0.0033 and E) brain (cortex and hippocampus) F_(1, 15)_= 5.031, p=0.0404. Abbreviations: KikGR, Kikume Green-Red (KikGR) photoconvertible fluorescent protein; CCI, controlled cortical impact.

We previously have shown that depletion of gut microbiota after TBI reduced infiltration of T cells into the brain which was associated with a pro-inflammatory microglial phenotype, reduced neurogenesis, and increased neurodegeneration(13, 33). These findings led us to pursue T cells as an important mechanistic link between the gut and the brain. To study the contribution of the intestinal T cell population that can traffic to the brain after TBI, we used KikGR33 transgenic mice which express a Kikume Green-Red (KikGR) photoconvertible fluorescent protein that undergoes stable photoconversion from a green to a red form after exposure to violet light(32). We illuminated the distal SI (6 cm of ileum) for 20 mins obtaining an effective KikG to KikR conversion in gut-resident lymphocytes (CD45^hi^CD11b^−^) and a minimum amount in the blood (Figure S1). Immediately after photoconversion, mice underwent sham or CCI (Fig. 1A), and we tracked the lymphocytes in the SI, blood, brain (Fig. 1B–E), and meninges (Figure S2) 7 days later by spectral flow cytometry. No significant differences were found between non-photoconverted vs photoconveted cells in the sham groups. However, we found a significant increase in the frequency of KikR + lymphocytes in the SI (Fig. 1B and C) and blood (Fig. 1B and D) 7 days after injury compared with mice without photoconversion or sham. KikR + lymphocytes were present in the ipsilateral injured brain compared with mice without photoconversion or sham (Fig. 1B and E) but not in the meninges (Figure S2) indicating that intestinal lymphocytes traffic to the brain parenchyma after injury(34, 35). These findings suggest that intestinal lymphocytes and trafficking are exacerbated by TBI. Although the myeloid cell population (CD45^hi^CD11b^+^) was also significantly photoconverted in the SI and detected in the blood (Figure S3B and C), we did not find KikR + myeloid cells in the brain (Figure S3D) indicating a specific contribution for gut-derived T cells to the brain at 7 days after TBI. However, we cannot discount that myeloid cells enter in the brain at earlier time points post-injury(3).

### Depleting T cells before an injury counteracts the effects of gut microbiota depletion on neurogenesis and reduces microglial activation.

**Figure 2:**
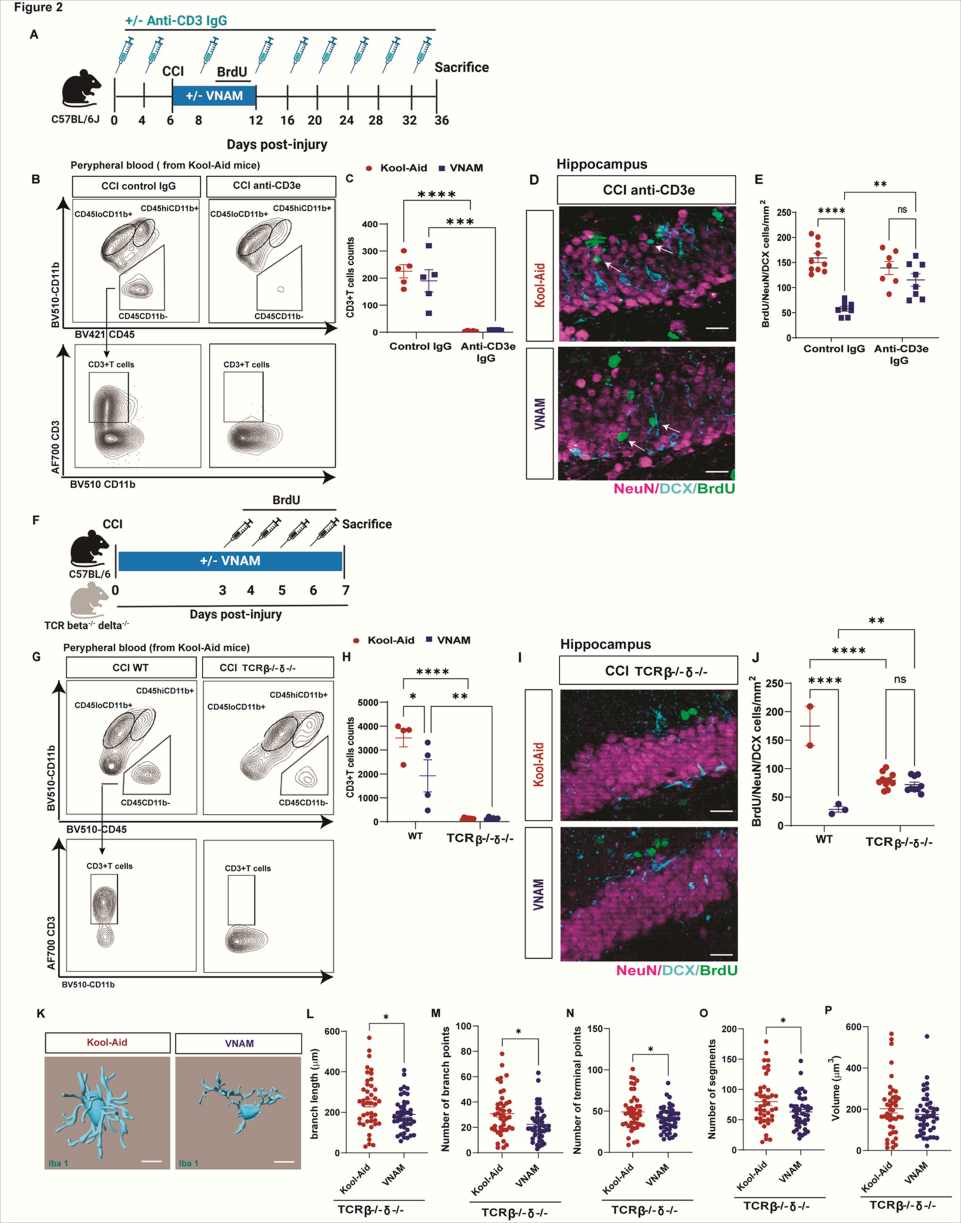
Depleting T cells before an injury counteracts the effects of gut microbiota depletion on neurogenesis and reduces microglial activation. A) 8-week-old mice were randomized to anti-CD3e or control IgG. Mice receive IP injections 6 and 2 days prior injury and every 4 days after injury starting on post-injury Day#2. Mice underwent CCI and were them randomized to Kool-Aid or VNAM in drinking water for 1 week. BrdU was injected in the last 4 days of VNAM administration. Mean values are plotted ± SEM, two-way ANOVA followed by Tukey’s multiple comparisons. F statistic for antibiotics*CD3 depletion presented unless otherwise noted (*p < 0.05, **p < 0.01 ***p < 0.001, ****p < 0.0001), n = 4–6 per group. B) Representative dot plots of gating strategy of CD3+T cells from a Kool-Aid mouse. C) Quantification of CD3+T cells in the blood _FCD3 depletion (1,16)_= 69.83, p<0.0001. D) Representative images of injured brain of BrdU/NeuN/DCX positive cells. Scale bar 20 μm. E) Density of BrdU/NeuN/DCX positive cells in the ipsilateral hippocampus F_antibiotics (1,29)_= 7.128, p=0.0123. F) Experimental design TCRβ^−/−^TCRδ ^−/−^ mice 7 days survival. G) Representative dot plots of gating strategy of CD3+T cells from a Kool-Aid mouse. H) Quantification of CD3+T cells in the blood F_(1, 18)_= 9.723 p=0.0059. I) Representative images of injured brain of BrdU/NeuN/DCX positive cells. Scale bar 20 μm. J) Density of BrdU/NeuN/DCX positive cells in the ipsilateral hippocampus F_(1, 20)_= 66.17, p<0.0001. E) Representative images of 3D microglia (Iba1+cells) reconstruction of Kool-Aid and VNAM groups. Microglia morphology analysis of L) dendrite length, M) branch points, N) terminal points, and O) volume. Mean values are plotted ± SEM, unpaired t-test *p < 0.05, n = 43–44 cells per group. Abbreviations: CCI, controlled cortical impact; VNAM, vancomycin, ampicillin, neomycin, and metronidazole; DCX, doublecortin; BrdU, Bromodeoxyuridine; TCR, T cell receptor.

Neurogenesis in the hippocampus increases after TBI, as the brain attempts to repair and regenerate following injury in mice(36) and humans(37). In the setting of neuroinflammation, activated T cells can participate in adult hippocampal neurogenesis once they infiltrate the brain(38). We previously reported that neurogenesis is significantly impaired after TBI in the setting of gut microbiota depletion(13, 33). We thus decided to study the role of T cells in neurogenesis in the context of GMD after TBI. To achieve this, we employed pharmacological T cell depletion with anti-CD3 IgG (Fig. 2A) prior to injury. To induce GMD, mice underwent antibiotic exposure (vancomycin, ampicillin, neomycin, and metronidazole, VNAM) in the drinking water for 7 days post-injury using Kool-Aid alone as a control group (Fig. 2A). Peripheral blood flow cytometry analysis following T cell depletion revealed a near-complete absence of CD3 + T cells (Fig. 2B and C). In the ipsilateral hippocampus we found that the density of neuronal lineage (NeuN or DCX) BrdU^+^ cells (Fig. 2D and E) were restored after the depletion of T cells in antibiotic-treated mice compared with control IgG injections. Furthermore, TCRβ^−/−^TCRδ^−/−^ mice (absence of alpha beta T cell receptor and any gamma delta T cell receptor) were exposed to VNAM for one week (Fig. 2F). Peripheral blood flow cytometry analysis of TCRβ^−/−^TCRδ^−/−^ mice revealed a nearly complete absence of CD3 + T cells (Fig. 2G and H). We observed that neurogenesis was unaffected by antibiotic depletion in TCRβ^−/−^TCRδ^−/−^ mice (Fig. 2I and J). Together these data provide evidence that T cells may be an important mechanistic link in gut microbial modulation of neurogenesis after trauma.

Given the key role of T cells in microglia development(39) and how TBI with GMD altered microglia activation(13), we wanted to further explore microglial phenotype in TCRβ^−/−^TCRδ^−/−^ mice 7 days post-injury with GMD. In the absence of T cells, microglia showed changes in morphology towards more amoeboid phenotype with a lower branch length (Fig. 2L), number of branch points (Fig. 2M), number of terminal points (Fig. 2N), and number of segments (Fig. 2O). However, we did not see changes in volume (Fig. 2P) between the groups, suggesting GMD-induced changes in microglial morphology are not as pronounced in the absence or depletion of T cells as we have reported previously in WT mice(13).

### SCFAs induced brain epigenetic changes without altering bacterial population.

**Figure 3:**
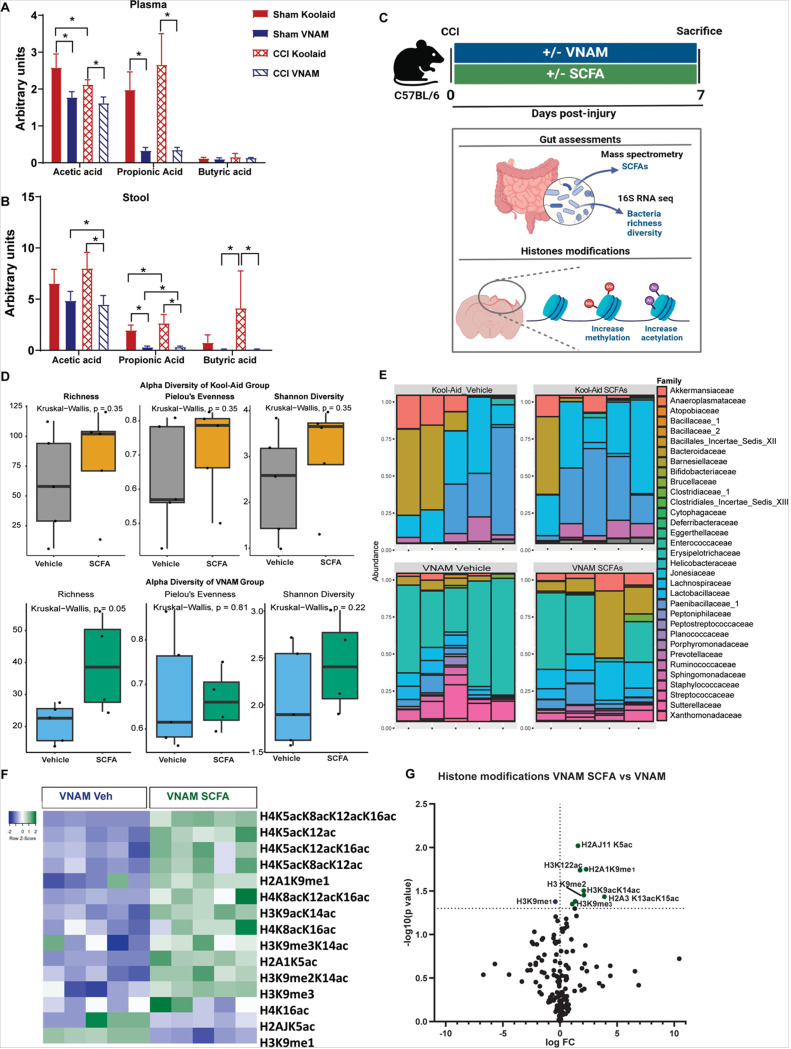
SCFAs induced brain epigenetic changes without altering bacterial population. A) Plasma and B) stool measurements of SCFAs via liquid chromatography one week after injury. C) Experimental strategy where mice were treated with and without VNAM supplemented or not supplemented with the mix of the most frequent SCFAs: acetate, butyrate, and propionate for 7 days post TBI. D) Graph depicts of richness, Pielous’s evenness, and Shannon a-diversity index of grouped data. E) Family-level phylogenetic classification of fecal 16S rDNA gene frequencies from before euthanasia (Day 7). Each bar represents an individual animal. Only families with a frequency >5% were included. F) Heat map of selected histones modifications in the brain of VNAM-treated mice with and without SCFA supplementation. G) Log_2_FC – log_10_P volcano plot of all detected histone post-translational modifications in the brain of VNAM-treated mice with or without SCFAs. Green dots depict P < 0.05 in the and black color indicate no significant differences comparing SCFAs treatment vs no treatment in the brain. Abbreviations: CCI, controlled cortical impact; VNAM, vancomycin, ampicillin, neomycin, and metronidazole; SCFAs, short-chain fatty acids.

SCFAs (acetate, butyrate, and propionate) are gut-derived metabolites produced by bacterial fermentation of the dietary fiber in the colonic lumen(19). SCFAs are important regulators of immunity, apoptosis, inflammation, and lipid metabolism in the gut(40). Importantly, their influence extends beyond the gut to other organs such as the brain(18). However, little is known about the effect that SCFAs have on TBI severity and recovery. To study the possible impact that gut bacteria depletion after TBI has on SCFA levels, we analyzed the concentration of SCFAs by mass spectrometry in both plasma (Fig. 3A) and stool (Fig. 3B) 7 day after TBI with and without antibiotics exposure. We observed a reduction of acetate and propionate in plasma after antibiotic exposure in sham and CCI mice (Fig. 3A). In addition, we detected a significant reduction of all three SCFAs in the stool of mice under GMD compared with Kool-Aid mice (Fig. 3B). The reduction of SCFAs in the intestine after antibiotic exposure led us to investigate the therapeutic impact that SCFA supplementation could have on TBI with GMD. We supplemented injured mice with acetate, butyrate and propionate for 7 days with and without antibiotic administration (Fig. 3C). To study whether SCFAs impacted gut bacteria population, we characterized the global pattern of microbial composition by 16S rRNA gene sequencing analysis of the fecal samples. We observed a subtle increase in richness, but no differences were found in Pieloús evenness or Shannon diversity after SCFA administration in the VNAM groups (Fig. 3D and E) and no significant changes were found between the Kool-Aid groups (Fig. 3D and E). SCFAs impact cellular functions and modulate immune responses by affecting gene expression and the epigenome through histone deacetylase (HDAC) inhibition(41), resulting in increased histone H3 acetylation(42). To characterize whether SCFAs induced epigenetic changes in the injured brain, we analyzed histone modifications by mass spectrometry 7 days post injury. Overall, we found significant changes in the enrichment of histone modifications after SCFA treatment compared with VNAM alone (Fig. 3F). We identified a significant increase in trimethylated H3Lys9 (H3K9m3), required in T cell lineage determination, muscle development and neurogenesis(43, 44), and acetylated histone H3 Lys9 and 14 (H3K9acK14ac), which plays a key role in neuron development and neurogenesis(23), in SCFA-treated mice in comparison with VNAM-veh (Fig. 3G). Taken together, these data suggest a crucial effect of SCFAs on immune cells and neuron differentiation through gene regulation after TBI with GMD, independent of bacterial community alterations. These findings raise the important mechanistic question of how these bacterial metabolites modulate the brain after TBI. One possible explanation is through T cells(45, 46).

### SCFAs rescue T cell differentiation and trafficking to the brain at 1-week post-injury with GMD.

**Figure 4:**
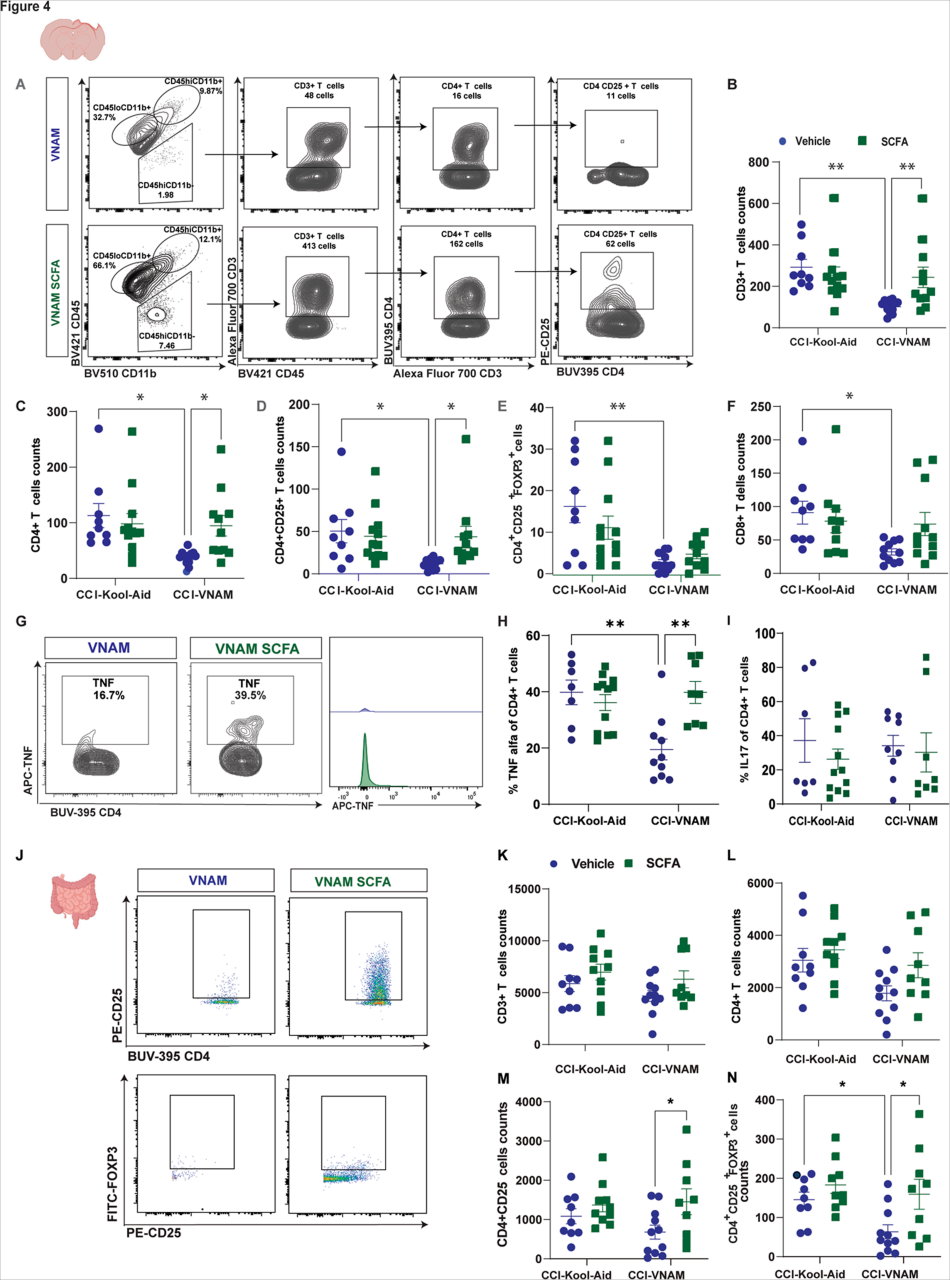
SCFAs rescue T cell differentiation and trafficking to the brain at 1-week post-injury with GMD. A) Flow cytometric gating strategy of the brain (cortex + hippocampus) of 7 days post-injury with GMD with and without SCFAs supplementation. Mean values are plotted ± SEM, two-way ANOVA followed by Newman-Keuls’multiple comparisons. F statistic for antibiotics*SCFAs treatment presented unless otherwise noted (*p < 0.05, **p < 0.01), n = 9–13 per group. Quantification of cell absolute numbers in the injured brain B) CD3+T cells F_(1, 41)_=6.300, p=0.0161. C) CD4+T cells, F_(1, 41)_= 5.055, p=0.0300. D) CD4^+^CD25+T cells, F_antibiotics(1, 41)_=4.164, p=0.0478. E) CD4^+^CD25^+^FoxP3+T cells, F_antibiotics(1, 41)_=5.032, p=0.0304. F) CD8+T cells F_antibiotics(1, 41)_=4.773, p=0.0347. G) Flow cytometry gating strategy and histogram of percentage of TNFα from CD4+T cells. H) Frequency of TNFα from CD4+T cells F_(1, 33)_=10.6, p=0.0026 and I) frequency of IL17 from CD4+T cells. J) Flow cytometric gating strategy of the small intestine (lamina propia) n = 9–11 per group. Quantification of cell absolute numbers in the lamina propia, K) CD3+T cells, L) CD4+T cells, M) CD4^+^CD25+T cells F_SCFAs(1, 35)_=5.763, p=0.0218, N) CD4^+^CD25^+^FoxP3+T cells F_antibiotics(1, 34)_ = 4.473, p=0.0418, F_SCFAs(1, 34)_ = 7.106, p=0.0117. Abbreviations: CCI, controlled cortical impact; VNAM, vancomycin, ampicillin, neomycin, and metronidazole; SCFAs, short-chain fatty acids.

SCFAs are crucial for T cell, especially T reg, differentiation, function, and proliferation in the colon, promoting colonic homeostasis and health(47–49). However, the role of SCFAs in the T cell response after brain trauma is unknown. In our previous work, we found that TBI increased immune cell infiltration into the brain and acute antibiotic depletion of the gut microbiota after TBI reduced peripheral immune cell infiltration(13). We hypothesized that SCFAs may be an important modulator of differentiation and/or trafficking of intestinal T cells to the brain after TBI. Thus, we aimed to study the impact of SCFA supplementation on T cell differentiation after TBI with GMD. We performed flow cytometry in the injured brain, blood, and SI (Lamina propria, LP) 7 days after TBI with GMD. Flow cytometry of cortical plus hippocampal cells in mice supplemented with SCFAs (Fig. 4A) revealed a reversal of reduced CD3 + T cells (Fig. 4B), CD4 + T cells (Fig. 4C), and CD4 + CD25 + T cells (T reg, Fig. 4D) brain infiltration associated with GMD. No changes were found in the CD4 + CD25 + FoxP3 (Fig. 4E) or CD8 + T cell (Fig. 4F) populations. As T cells undergo differentiation, they increase their capacity to release cytokines, while progressively losing their capacity to proliferate(50). We found that SCFAs induced intracellular TNFα expression in CD4 + T cells (Fig. 4G and H). No changes were found in Th17 cells (intracellular expression of IL17, Fig. 4I). Our data support the hypothesis that SCFA supplementation not only increased T cell infiltration into the brain but restored the proportion of specific T helper (Th) subsets in the brain after TBI. We performed flow cytometry of the intestinal LP but found no major changes in total the T helper cell population (Fig. 4J–L). However, SCFA supplementation induced a significant increase in T reg total counts (Fig. 4M) as well as maturation status (CD4 + CD25 + FoxP3 T cells, Fig. 4N). No changes were found in the proportion of Th cells in the SI or blood (Figure S4) indicating that SCFAs specifically may influence the differentiation or infiltration of certain T cell subsets within the brain itself.

**Figure 5:**
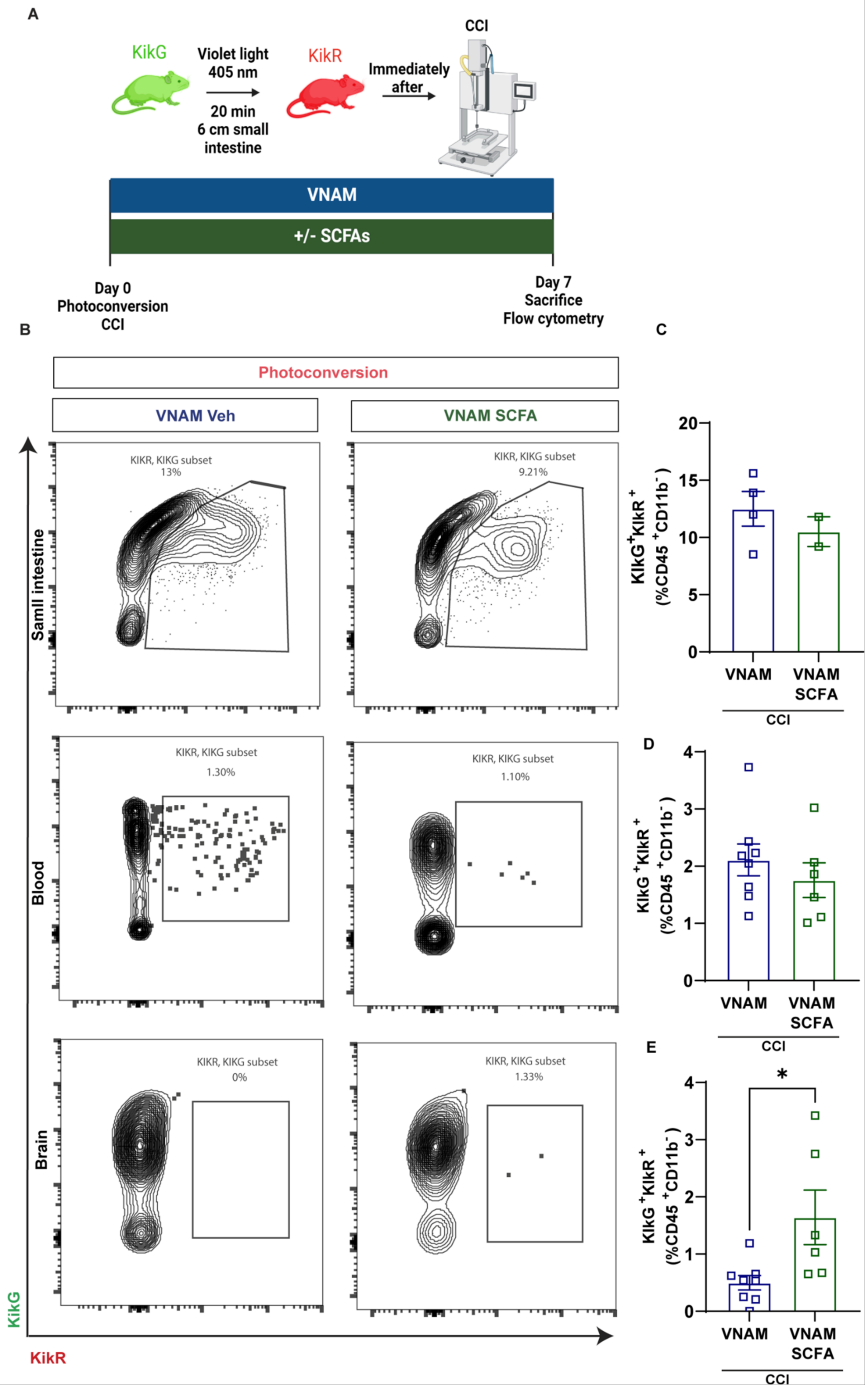
Intestinal T cells infiltration and differentiation into the brain 7 days after TBI with GMD. A) Experimental design for analysis of KikGR mice. Photoconversion of the distal part of the small intestine is induced using violet light right before CCI, and the photoconverted lymphocytes (CD45^+^CD11b^−^KikR^+^) cells are analyzed in SI, blood, and brain by spectral flow cytometry 7 days after CCI with GMD with or without SCFAs supplementation. B) Flow cytometric gating strategy in photoconverted (KikR^+^) mice of SI, blood and brain. Lymphocytes (CD45^+^CD11b^−^) that expresses the red form (KikR^+^) cells 7 days after CCI. C) Frequency of photoconverted lymphocytes (CD45^+^CD11b^−^KikR^+^) in the small intestine (lamina propia), D) blood and E) brain (cortex and hippocampus). Mean values are plotted ± SEM, unpaired t-test *p < 0.05, n = 6–8 per group. Abbreviations: KikGR, Kikume Green-Red (KikGR) photoconvertible fluorescent protein; CCI, controlled cortical impact; VNAM, vancomycin, ampicillin, neomycin, and metronidazole; SCFAs, short-chain fatty acids.

Next, utilizing KikGR33 mice, we evaluated the impact of SCFAs on intestinal T cell trafficking after TBI with GMD. We exposed and illuminated SI, then mice were given antibiotics with or without SCFA supplementation for 7 days post-injury (Fig. 5A). To characterize changes in intestinal immune cell trafficking associated with SCFA supplementation, we used spectral flow cytometry to analyze the percentage of lymphocytes (CD45^hi^CD11b^−^ cells) that were photoconverted from green to red in the SI, blood, and brain. We did not find differences in the percentage of photoconverted lymphocytes in the SI (Fig. 5B and C) or blood (Fig. 5B and D). However, we observed that SCFA supplementation after injury enhanced the trafficking of intestinal lymphocytes to the brain (Fig. 5B and E). No changes were found in the trafficking of monocytes from the gut to the brain (Figure S5). We have demonstrated that SCFAs enhance gut T cell trafficking to the brain inducing either a specific T cell differentiation once they infiltrated the brain parenchyma 7 days post-injury with GMD or a selective infiltration of differentiated T cells. Future investigations are required to delineate between these two possibilities. Together, these data support SCFAs as gut-derived mediators of T cell differentiation and intestinal trafficking to the brain after trauma.

### SCFAs restore GMD induced reductions in post-TBI neurogenesis in a T cell dependent manner.

**Figure 6:**
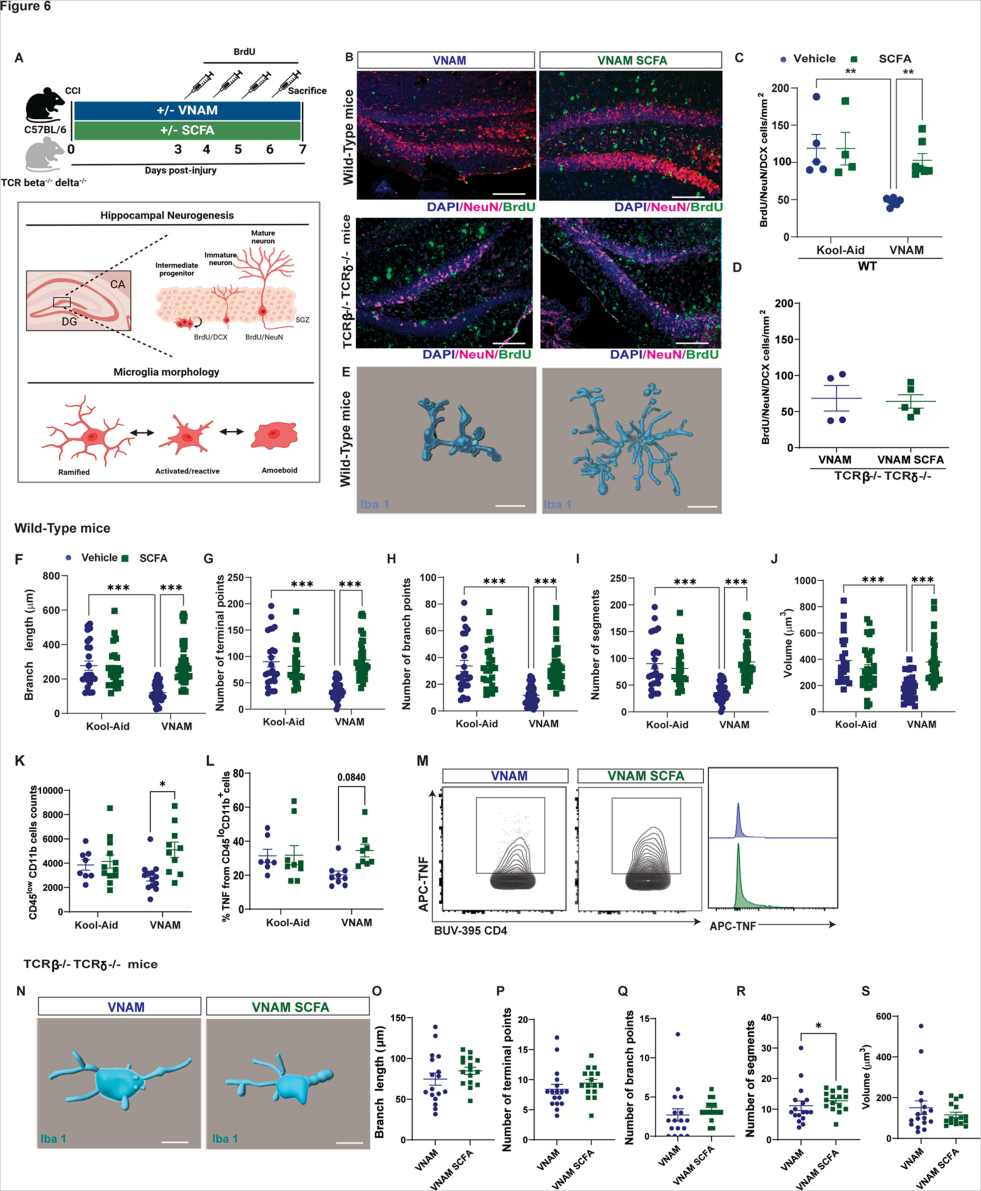
SCFAs restore microglia morphology and neurogenesis at 1-week post-injury with microbiota depletion in a T cell-dependent manner. A) Experimental design for analysis of neurogenesis and microglia morphology 7 days after TBI with GMD with and without SCFAs supplementation. B) Representative images of injured hippocampus of DAPI/NeuN/BrdU positive cells. Mean values are plotted ± SEM, two-way ANOVA followed by Tukey’s multiple comparisons. F statistic for antibiotics*SCFAs presented unless otherwise noted (*p < 0.05, **p < 0.01), n = 4–6 per group. C) Density of BrdU/NeuN/DCX positive cells in the ipsilateral hippocampus in wild type mice F_(1, 19)_=5.15, p=0.0350, n=4–7, and in D) TCRβ^−/−^TCRδ^−/−^ mice, n=4–5. Mean values are plotted ± SEM, unpaired t-test *p < 0.05, n = 6–8 per group. E) Representative images of 3D microglia (Iba1+cells) reconstruction of VNAM and VNAM SCFA in the injured hippocampus. Scale bar: 50 μm. Mean values are plotted ± SEM, two-way ANOVA followed by Tukey’s multiple comparisons. F statistic for antibiotics*SCFAs presented unless otherwise noted (*p < 0.05, **p < 0.01 ***p < 0.001, ****p < 0.0001), n = 32–35 per group. F) Imaris analysis of microglia morphology of branch length F_(1, 112)_=19.08, p<0.0001, G) terminal points F_(1, 112)_=23.89, p<0.0001, H) branch points F_(1, 112)_=16.76, p<0.0001, I) segments F_(1, 112)_=23.89, p<0.0001 and J) volume F_(1, 112)_=20.46, p<0.0001. Scale bar: 30 μm. K) Flow cytometric gating strategy of TNFα+microglia cells and TNFα+microglia histogram. L) Total counts of microglia (CD45^lo^CD11b^+^ cells) F_(1, 41)_=5.508, p=0.0238. M) Frequency of TNFα from microglia F_SCFAs(1, 29)_=3.127, p=0.0875. N) Representative images of 3D microglia (Iba1+cells) reconstruction of VNAM and VNAM SCFA in the TCRβ^−/−^TCRδ^−/−^ injured hippocampus. Imaris analysis of microglia morphology of O) branch length, P) number of terminal points, Q) number of branch points, R) number of segments and S) volume. Scale bar: 30 μm. Mean values are plotted ± SEM, unpaired t-test *p < 0.05, n = 17–16 microglia per group. Abbreviations: CCI, controlled cortical impact; VNAM, vancomycin, ampicillin, neomycin, and metronidazole; SCFAs, short-chain fatty acids, DCX, doublecortin; BrdU, Bromodeoxyuridine; TCR, T cell receptor. Created in BioRender. Steed, A. (2024) BioRender.com/q19s665.

SCFAs are also thought to be involved in modulating hippocampal neurogenesis(51) and microglia phenotype(18) in heath and disease. Considering our results that SCFAs enhanced T cell infiltration, differentiation, and trafficking in gut microbiota depleted mice, we investigated the impact of SCFAs on neurogenesis and microglial response in GMD and TBI mice 7 days post-injury in the presence and absence of T cells (Fig. 6A). We found SCFAs rescued GMD-impaired neurogenesis after injury (Fig. 6B and C), implicating these metabolites as possible mediators of gut microbiota modulation of TBI-induced neurogenesis. Furthermore, in the absence of T cells, SCFAs no longer rescued neurogenesis in antibiotic-treated mice (Fig. 6B and D). We further characterized microglial morphology and observed a more ramified morphology in antibiotic depleted mice supplemented with SCFAs (Fig. 6E) with an increase in branch length (Fig. 6F), number of terminal points (Fig. 6G), number of branch points (Fig. 6H), number of segments (Fig. 6I), and volume (Fig. 6J). In addition, by flow cytometry, we observed an increase in number of microglia (Fig. 6K, CD45^lo^CD11b^+^ cells) as well as microglia TNFα + cells (Fig. 6L and M). When we analyzed the microglia morphology (Fig. 6N) in TCRβ^−/−^TCRδ^−/−^ mice treated with antibiotics we did not find significant changes in branch length (Fig. 6O), number of terminal points (Fig. 6O), number of branch points (Fig. 6Q), or volume (Fig. 6S) with a mild increase in the number of segments in SCFAs supplemented mice. Those data suggest that T cells are partially required for SCFA-rescued microglial morphological changes in the setting of TBI with GMD. Moreover, T cells are essential for SCFA-restored neurogenesis in the setting of TBI with GMD.

### SCFAs induce T cell infiltration through FFA2 but not FFA3 receptor activation.

**Figure 7:**
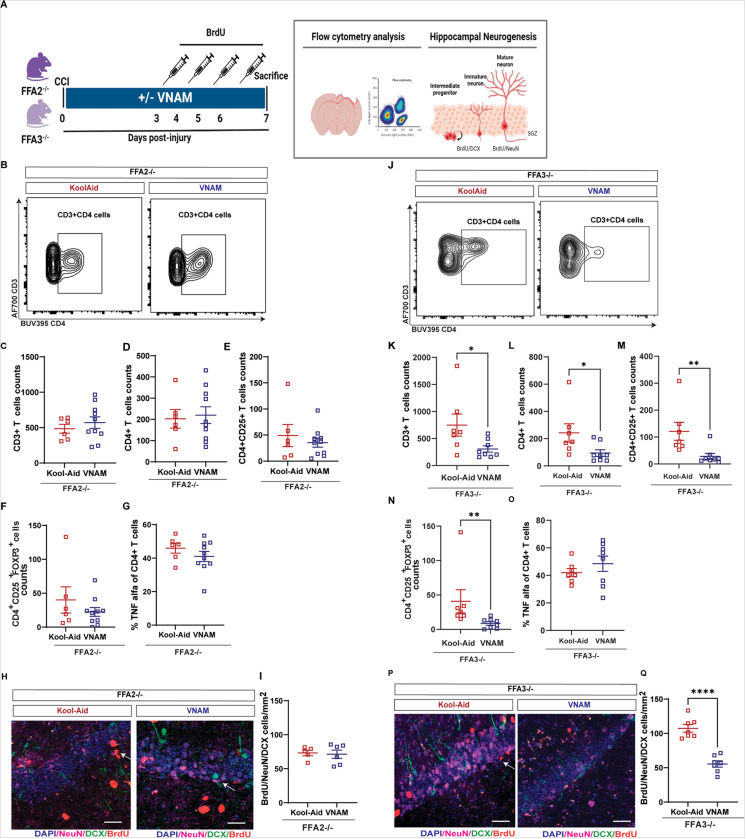
Gut-brain communication by T cells is FFA2-dependent. A) Experimental design of flow cytometry analysis and neurogenesis 7 days after TBI with GMD using *Ffa2*^−/−^ and *Ffa3*^−/−^ mice. B) Flow cytometric gating strategy of the brain (cortex + hippocampus) of 7 days post-injury with or without GMD of the *Ffa2*^−/−^. Quantification of cell absolute numbers in the *Ffa2*^−/−^ injured brain C) CD3+T cells; D) CD4+T cells; E) CD4^+^CD25+T cells; F) CD4^+^CD25^+^FoxP3+T cells; G) Frequency of TNFα from CD4+T cells. H) Representative images of *Ffa2*^−/−^ injured hippocampus of DAPI/NeuN/BrdU positive cells. Scale bar: 20 μm. I) Density of BrdU/NeuN/DCX positive cells in the *Ffa2*^−/−^ ipsilateral hippocampus. J) Flow cytometric gating strategy of the brain (cortex + hippocampus) of 7 days post-injury with or without GMD of the *Ffa3*^−/−^. Quantification of cell absolute numbers in the *Ffa3*^−/−^ injured brain K) CD3+T cells; L) CD4+T cells; M) CD4^+^CD25+T cells; N) CD4^+^CD25^+^FoxP3+T cells; O) Frequency of TNFα from CD4+T cells. P) Representative images of *Ffa3*^−/−^ injured hippocampus of DAPI/NeuN/BrdU positive cells. Scale bar: 20 μm. Q) Density of BrdU/NeuN/DCX positive cells in the *Ffa3*^−/−^ ipsilateral hippocampus. Mean values are plotted ± SEM, unpaired t-test ****p < 0.0001, n = 6–7 per group. Abbreviations: CCI, controlled cortical impact; VNAM, vancomycin, ampicillin, neomycin, and metronidazole; FFAR, free fatty acid receptors; DCX, doublecortin; BrdU, Bromodeoxyuridine.

One of the possible mechanisms for how SCFAs alter T cell responses is via activation of cell surface receptors GPR41 (FFA3) and GPR43 (FFA2)(52). Studies have shown that FFA3 stimulation induced by acetate or propionate impairs Th2 responses(52). In addition, FFA2 stimulation is required for acetate or propionate to promote colonic T reg expansion during gut inflammation(48). Thus, we aimed to determine whether FFA2 and/or FFA3 were required for T cell differentiation and infiltration to the brain after TBI with GMD. We used global FFA2 and FFA3 knock-out mice with or without antibiotic-induced GMD for 1-week post-injury (Fig. 7A). Conventional flow analysis in *Ffa2*^−/−^ mice showed that the infiltration of the total counts of CD3 + T cells (Fig. 7C), CD4 + T cells (Fig. 7D), CD4 + CD25 T cells (Fig. 7E), CD4 + CD25 + FoxP3 T cells (Fig. 7F) and of the percentage of Th1 (Fig. 7G, CD4 + TNFα) lymphocytes was unaffected by antibiotic depletion after TBI as seen in the WT mice (Fig. 4). These results were associated with no changes in neurogenesis between the groups (Fig. 7H and I). Surprisingly, in *Ffa3*^−/−^ mice (Fig. 7J) there was a decrease of the total counts of CD3 + T cells (Fig. 7K), CD4 + T cells (Fig. 7L), and CD4 + CD25 T cells (Fig. 7M), and CD4 + CD25 + FoxP3 T cells (Fig. 7N) but no changes were observed on the percentage of Th1 (Fig. 7O, CD4 + TNFα) lymphocytes as previously described in WT mice (Fig. 4). These results were associated with significant reductions in neurogenesis in antibiotic-exposed mice compared with Kool-Aid-treated mice (Fig. 7P and Q). These findings support the hypothesis that FFA2, but not FFA3, is required for T cell infiltration and differentiation in the brain and neurogenesis in the setting of TBI with GMD. SCFAs are potent modulators of both T cell responses and possibly orchestrate the crosstalk between the gut and the brain by FFA2-related signaling.

## Discussion

Our findings demonstrate that T cells are a cellular link required in gut-brain communication after TBI. Furthermore, we observed that SCFAs, produced by the microbiota, can modulate T cell trafficking and differentiation in an FFA2-dependent manner associated with improvement of neurogenesis and reduced microglia activation. We previously described that microbial depletion immediately after TBI decreased the infiltration of lymphocytes in the brain over time and was associated with worsened outcomes after TBI(13). In the present study, we have further observed that intestinal T cells are part of the pool of the lymphocytes that traffic to the brain after TBI. Supplementation of SCFAs to mice with disrupted gut microbiota and subjected to TBI increased intestinal T cell trafficking to the brain, and increased the brain population of T reg cells, which have an important role in regulating inflammation along the gut-brain axis after TBI(53). In addition, we observed restored neurogenesis and microglia homeostasis to levels seen in injured mice with intact microbiota. Our findings support T cells as an essential mediator by which the gut microbiota modulates TBI pathogenesis and highlight new opportunities to target neuroinflammation and enhance TBI recovery.

Increasingly T cells are recognized to play a key role in the regulation of immune responses during secondary injury after brain injury such as TBI(9, 10), stroke(12) and spinal cord injury(54), impacting disease progression and recovery(55). After stroke, *Rag1*^−/−^ mice, which lack T cells and B cells, show decreased lesion volumes and neurological deficits compared with injured WT mice, with these outcomes reversed after T cell repopulation(56, 57). Moreover, pharmacologically inhibiting T cell infiltration following a stroke has been linked to reduced infarct size and improved outcomes(58). After traumatic CNS injuries, including facial nerve transection, TBI, and optic nerve crush injury, T cells can induce both detrimental and/or beneficial effects. In TBI, it was demonstrated that brain CD8 + T cells contributed to chronic motor deficits and myelin pathology and deficiency/depletion of CD8 + T cells promoted neurological recovery(10). In optic nerve crush injury or spinal cord injury, IL-4-producing T cells induced neuroprotective effect in an antigen-independent manner(54). We found that intestinal T cells are part of the pool of lymphocytes that infiltrate the brain after TBI. In addition, SCFA supplementation induced T cell trafficking, implicating intestinal metabolites in T cell responses. We also described that T cells were required for GMD-induced microglial and neurogenesis alterations after TBI. We observed that in the absence of T cells, neurogenesis was completely unimpacted by antibiotic exposure after TBI. Therefore, these data support that intestinal T cells are part of the pool of lymphocytes in the injured brain and may be required for the gut-brain interaction in the setting of TBI with GMD.

SCFAs have been reported to impact gastrointestinal immune cells, specifically T reg cells(48, 59), T effector cells(60), and macrophages(61) in the gastrointestinal tract. However, the impact of SCFAs on T cell trafficking and differentiation in the injured brain is not fully understood. T reg cells are regulators of inflammation and have shown to protect the brain against pathological inflammation after CNS injury(62). Systemic T reg depletion after TBI has been shown to be associated with exacerbation of motor deficits, reactive astrocytes and increased IFNγ expression(63). In our previous work, we found decreased infiltration of T reg cells after TBI in mice with GMD(13, 33). In the present manuscript, we report that SCFA supplementation following TBI in mice with GMD led to an increase in regulatory T cells (T regs, CD4 + CD25 + T cells) in both the brain and intestine suggesting their possible essential role in the gut-brain communication after TBI. The observed reduction in microglial activation and the enhancement of neurogenesis may be attributed to this increase in T regs and their immunosuppressive capabilities. By promoting a more anti-inflammatory environment, T regs may help reduce neuroinflammation, thereby facilitating the recovery processes. However, further studies are needed to validate these associations, particularly through T reg depletion approaches.

We observed that SCFA supplementation did not produce any noticeable effects on TBI outcomes, such as alteration in neurogenesis, T cells response or microglia activation, when administered to mice without prior antibiotic exposure. One possible explanation is due to mild SCFA concentration changes at 7 days post-injury. We observed significant differences in the levels of acetate but not on butyrate and propionate in plasma at 7 days post-injury. In another report, fecal acetate concentrations were mildly decreased at 7 days with lower levels 28 days post-injury, suggesting that SCFA concentrations continued to decrease over time(17). Therefore, the potential for an early therapeutic effect for SCFA supplementation early after TBI may be limited but more chronic administration may have a therapeutic effect.

One of our main observations was that SCFAs enhanced intestinal T cells trafficking to the brain but not intestinal myeloid cells highlighting the importance of SCFAs-T cells interaction within the gut-brain axis. These findings were associated with an increased the percentage of TNFα expressing CD4 + T cells in the brain. TNFα is thought of as a pro-inflammatory cytokine, however recent findings by others have demonstrated that TNFα2 receptor activation is required for expansion and differentiation of T regs and their anti-inflammatory benefits in autoimmune diseases(64). Additionally, the TNFα2 receptor is required for TNFα-induced leukocyte-endothelial interaction *in vivo*(65, 66). Further investigations are required to characterize the role of TNFα2 receptor-TNFα pathway in the endothelial cell in the setting of TBI-GMD under SCFA supplementation.

SCFAs administration reversed the microglial changes seen in uninjured GF mice despite the absence of SCFA receptors (G-protein coupled receptor 43, GPR43, also known as FFA2) expression in any neuroectodermal CNS cell type(18). Our present study extends this previous work showing that SCFAs induced changes in microglia morphology and TNFα expression in our TBI model only in the presence of GMD. These findings raise the important mechanistic question of how these bacterial metabolites modulate microglia overcoming the BBB, dilutional challenges, and the lack of FFA2 and FFA3 expression. One possible explanation is through T cells. Recently, brain-resident CD4 + T cells have been identified as having an important role in microglia maturation(39). Interestingly, in a murine stroke model with antibiotic depletion of the gut microbiota, SCFAs supplementation altered the microglial response, but in the absence of T cells (using Rag1^−/−^ mice) the SCFAs effect on microglia was no longer observed(67). In this present manuscript, we described how T cells are required for microglia activation in TBI with GMD. In addition, after SCFA supplementation, in the absence of T cells, we did not observe SCFA-restored microglia homeostasis after TBI with GMD implying that T cells play an important role in the communication between the gut microbiota and microglia in an injured brain.

T cell response is regulated by several factors such as infection or injury and autoimmune diseases that can deliver antigens and cytokines regulating T cell differentiation into functionally specialized effector and regulatory(68). A growing body of evidence suggests that SCFAs have a profound impact on T cells differentiation in the gut, specifically among CD4 + T cell subsets(47). One of the mechanisms by which SCFAs mediate their effects is through biding FFA2 and FFA3. SCFA-FFA interaction profoundly affect inflammatory responses(69). T cells can be differentiated to T reg by HDAC inhibition in a FFA2-dependent manner(48). In light with these findings and considering our data that gut bacteria depletion after TBI directly influence T cell differentiation and infiltration and how SCFA reserved it, we asked if gut-brain communication by T cells was a receptor-dependent process. In absence of FFA2 but not FFA3, antibiotic depletion of the gut microbiota T cell infiltration was no longer reduced in the injured brain. Furthermore, in antibiotic-exposed mice adult hippocampal neurogenesis was reduced in WT and *Ffa3*^−/−^ mice but not in *Ffa2*^−/−^ mice. Hence, our data provides evidence that T cells are an essential mediator of gut to brain communication, especially impacting neurogenesis, after TBI in a FFA2 dependent manner. Nevertheless, we could not evaluate whether FFA2 were essential for T cell activation, differentiation and function in TBI with GMD. Further investigation is needed including cell-specific FFA2 deletion models. In addition, further studies are necessary to uncover the possible cellular and molecular mechanisms behind FFAs-SCFA interaction in T cell response that may represent new avenues for understanding and manipulation the immune response in the TBI settings.

### Limitations

Our studies do have some limited translatability to patients exposed to antibiotics early in their course of recovery after TBI. However, patients are generally exposed to intravenous antibiotics with a narrower spectrum than the antibiotics used in these studies(70). Additionally, we did not use an alternative approach of gut microbiota depletion or absence without direct exposure to antibiotics such as GF or fecal microbiota transplantation (FMT) models to control the possible confounding systemic affects antibiotic exposure. However, a previous publication from our group showed that GF mice receiving FMT from VNAM-treated mice demonstrated microglia activation, reduced infiltration of T cells, and decreased neurogenesis similarly to specific-pathogen-free antibiotic-depleted mice(33). Previous research has shown that SCFAs induce long-term spatial learning improvement up to 28 days port-TBI(17). Therefore, another limitation of this study is the lack of information regarding whether acute or chronic SCFAs treatment might influence long-term behavioral changes. Aware of this limitation, further investigation of the role of SCFAs in chronic neuroinflammation, neurodegeneration and memory lost in TBI with GMD is needed.

## Conclusions

To our knowledge, this is the first study to characterize that T cells subsets infiltration and/or differentiation into the brain after TBI is essential mechanism by which the gut microbiota modulates neuroinflammatory response, impacting neurogenesis and microglia activation. SCFAs are required for the T cell-induced gut-brain communication in a receptor-dependent manner. However, SCFA supplementation did not produce any noticeable effects on neurogenesis or neuroinflammation after TBI when administered to mice without antibiotic induced GMD. Therefore, further investigation of a deeper understanding of the mechanisms by which TBI associated neuroinflammation is modified by the gut microbiota will generate novel insights and opportunities to develop critically needed approaches to facilitate neuroprotection and highlight new opportunities to target neuroinflammation and enhance neuronal survival.

## Supplementary Material

Supplement 1

## Figures and Tables

**Table 1:** Overview of the primary antibodies used in the present study

Antibody	Fluorophore	Clone	Species	Dilution		Source	Product number

				Tissue	In vitro		

				IHQ	FC		

CD45	BV425	30-F11	Rat monoclonal		1:200	BioLegend	103134

CD45	PerCP	30-F11	Rat monoclonal		1:960	Cytek Biosciences	67-0451-U100

CD3	AF700	500-A2	Armenian Hamster monoclonal		1:100	BioLegend	100320

CD4	BUV395	GK1.5	Rat monoclonal		1:250	BD Biosciences	565974

CD8a	PerCP-Cy5.5	53 – 6.7	Rat monoclonal		1:200	BioLegend	100733

CD11b	BV510	M1/70	Rat monoclonal		1:500	BioLegend	101263

CD11b	PE-fire 810	M1/70	Rat monoclonal		1:1920	BioLegend	101285

CD25	PE	PC61	Rat monoclonal		1:200	BioLegend	102007

FOXP3	FITC	FJK-160	Rat monoclonal		1:100	Invitrogen	11-5773-80

TNFα	APC	MP6-XT22	Mouse monoclonal		1:100	BioLegend	506307

IL17a	PE-Cy7	TC11-18H10.1	Mouse monoclonal		1:100	BioLegend	506921

NeuN		A60	Mouse monoclonal	1:1000		Millipore	MAB377

NeuN		A60	Rabbit polyclonal	1:4000		Millipore	MAB377

DCX			Rabbit polyclonal	1:1000		Abcam	Ab18723

BrdU		BU1/75	Rat monoclonal	1:150		Abcam	Ab6326

Iba1		NCNP24	Rabbit polyclonal	1:1000		Wako	019-19741

Secondary antibody	AF594		Donkey anti-rat	1:500		Thermo Fisher	

Secondary antibody	AF647		Donkey anti-mouse	1:500		Thermo Fisher	

Secondary antibody	AF488		Donkey anti-rabbit	1:500		Thermo Fisher	

Secondary antibody			Biotinylated goat anti-rabbit	1:1000		Vector Laboratories	BA-1000-1.5

## Data Availability

The datasets generated and analyzed during the current study are available from the corresponding author on reasonable request.
