# Supplementary material for: Short-chain fatty acids are a key mediator of gut microbial regulation of T cell trafficking and differentiation after traumatic brain injury
Source: Res Sq. 2024 Nov 21:rs.3.rs-5397327. Preprint. [Version 1] doi: 10.21203/rs.3.rs-5397327/v1 (PMC11601855; doi:10.21203/rs.3.rs-5397327/v1)
Supplement: Supplement 1 [file NIHPPRS5397327V1-supplement-1.pdf]

Supplementary Files

This is a list of supplementary files associated with this preprint. Click to download.

- supplementalfigures.docx
